# Effect of feeding wood kraft pulp on the growth performance, feed digestibility, blood components, and rumen fermentation in Japanese Black fattening steers

**DOI:** 10.1111/asj.13182

**Published:** 2019-02-27

**Authors:** Yuka Maeda, Keiko Nishimura, Kazuhiro Kurosu, Hitoshi Mizuguchi, Shigeru Sato, Fuminori Terada, Shiro Kushibiki

**Affiliations:** ^1^ Miyazaki Livestock Research Institute Nishimorokata‐gun Miyazaki Japan; ^2^ Graduate School of Life and Environmental Sciences Tsukuba University Tsukuba Ibaraki Japan; ^3^ Nippon Paper Industries Co., Ltd. Tokyo Japan; ^4^ DKK‐TOA Yamagata Corporation Shinjo Yamagata Japan; ^5^ Faculty of Agriculture Iwate University Morioka Iwate Japan; ^6^ Faculty of Agriculture Tohoku University Sendai Japan; ^7^ Institute of Livestock and Grassland Science NARO Tsukuba Ibaraki Japan

**Keywords:** digestibility, growth performance, Japanese Black fattening steers, rumen fermentation, wood kraft pulp

## Abstract

This study aimed to examine the effects of feeding kraft pulp (KP) on the growth performance, feed digestibility, and rumen fermentation of Japanese Black fattening steers. Ten Japanese Black fattening steers (aged 26 months) were randomly divided into control and KP groups. The control group (*n* = 5) was fed concentrate feed without KP, and the KP group (*n* = 5) was fed concentrate feed containing 10% KP. Both the groups were provided rice straw as roughage. The experiment was conducted over a period of 12 weeks. There was no significant difference in dry matter intake, daily body weight gain, and nutrient digestibility between both groups. No difference was observed in the ruminal concentrations of volatile fatty acids among the groups. At weeks 8 and 12 after the onset of the experiment, the acetate‐to‐propionate ratio in the ruminal fluid of the KP group was significantly higher than that of the control group. The average daily pH of ruminal fluid and activity of ruminal lipopolysaccharide did not differ between the groups. Our results suggested that the growth performance and feed digestibility in the Japanese Black fattening steers were not influenced by replacing concentrate feed with KP.

## 1 INTRODUCTION

In Japan, the Japanese Black steers are normally fattened from the age of 9 months, for about approximately 20 months (Ministry of Agriculture, Forestry and Fisheries Japan (MAFF), 2015). The fattening period is divided into three stages: early, middle, and late. The roughage‐to‐concentrate ratio and the amount of vitamin A (VA) added in the feed vary with these stages (National Agriculture and Food Research Organization (NARO), 2009). For instance, the proportion of the concentrate feed is increased from approximately 55% (during the early period) to 85%–90% (in the late period) (Inoue, 2005). Additionally, a correlation between the decreased blood VA concentration and increased beef marbling standard number (Oka, Maruo, Miki, Yamasaki, & Saito, 1998), which affects the value of the beef, as observed during the middle fattening period, have led to the restriction of VA administration. Unique to the Japanese Black steer, this type of fattening aims to achieve a balance between development of the gastrointestinal tract and muscle and improvement of meat quality (Inoue, 2005).

An increased proportion of the concentrate feed promotes rumen fermentation and alters both the pH of the ruminal fluid and production of volatile fatty acids (VFAs) (Owens, Secrist, Hill, & Gill, 1998). Particularly, the oversupply of concentrate feed during fattening may lead to a daily decrease in the pH of ruminal fluid, thus increasing the risk of metabolic disorders. A sustained decrease in the pH of ruminal fluid in daily cattle is diagnosed as subacute ruminal acidosis (SARA). SARA is defined as ruminal fluid pH depression below 5.8 for more than 3 hr per day (Gozho, Plaizier, Krause, Kennedy, & Wittenberg, 2005). SARA causes reduced feed intake (Enemark Jorg, 2008; Plaizier, Krause, Gozho, & McBride, 2008), decreased fiber component digestibility (Guo et al., 2013), and liver dysfunction (Plaizier et al., 2008). Although SARA has not been reported in the Japanese Black fattening steer, a sustained decrease in the pH of ruminal fluid is assumed to induce metabolic disorders and to decrease productivity. Nutritional management of the Japanese Black steer during fattening is therefore necessary to control rumen fermentation without reducing the energy level of the feed, thereby improving the health and productivity of the animals.

Recently, wood kraft pulp (KP), which is high in total digestible nutrients (TDN) and neutral detergent fiber (NDF), has been developed as a feed. Kraft pulp is a feed that is primarily cellulose; it results from the selective removal of lignin, which is difficult to break down in the gastrointestinal tract, using alkaline treatment. Kraft pulp contains the same level of TDN as rolled corn, but it has an in vitro fermentation pattern which falls between that of concentrate feed and roughage (Hada, Yashro, Machida, & Kajikawa, 2016). When provided to the lactating cows as a substitute for rolled corn, KP contributes to stable rumen fermentation by increasing the pH of ruminal fluid and decreasing the activity of ruminal lipopolysaccharide (LPS) (Nishimura et al., in press). Providing KP to the Japanese Black fattening steer is expected to both stabilize the ruminal environment and improve productivity; however, to the best of our knowledge, no reports are available on the effect of feeding KP to the Japanese black fattening steer.

Therefore, this study aimed to investigate the effects of feeding KP on the growth performance, blood characteristics, rumen properties, and feed digestibility of the Japanese Black fattening steer.

## 2 MATERIALS AND METHODS

### 2.1 Animals and diets

Ten Japanese Black fattening steers (aged 26 months) were used in this study, and the experiment was conducted over a period of 12 weeks. The steers were housed in individual barns and had free access to fresh water and mineral blocks. The steers were randomly divided into control and KP groups. The control group (*n* = 5) was fed concentrate feed without KP, and the KP group (*n* = 5) was fed concentrate feed containing 10% on a dry matter (DM) basis KP, which was supplied by Nippon Paper Industries Co., Ltd (Tokyo, Japan). The ingredients and nutritional composition of the diets are presented in Table 1. In the KP group, the steers were initially fed concentrate, and the proportion of KP was gradually increased up to 10% from 4 weeks before the onset of the experiment.

**Table 1:** Ingredients and nutritional composition of the experimental diet

Parameter	Concentrate feed		Roughage
	Control	10% KP^a^	
Ingredient, % of DM			
Commercial formula feed^b^	100.0	89.5	—
KP^a^	—	10.0	—
Urea	—	0.5	—
Rice straw	—	—	100.0
Nutritional composition			
DM, %	87.5	88.5	86.0
OM, % of DM	96.8	97.2	80.8
CP, % of DM	13.9	13.9	4.5
EE, % of DM	3.2	2.8	1.4
aNDFom, % of DM	18.0	24.4	63.9
Starch, % of DM	53.2	49.2	5.2

*Notes*. DM, dry matter; OM, organic matter; CP, crude protein; EE, ether extract; aNDFom, α‐Amilastreated ash‐free neutral detergent fiber.

a Kraft pulp.

b Miyazaki shimofuri tokugou shiageyou, Minami nihon kumiai siryo.

The steers were fed concentrate and rice straw in a ratio of 110% TDN to that of the Japanese Feeding Standard for Beef Cattle (National Agriculture and Food Research Organization (NARO), 2009), with an expected daily weight gain (DG) of 0.75 kg. The roughage‐to‐concentrate ratio was 1:9. The steers were provided experimental diets twice daily at 09:00 and 16:00 hours. The orts from individual steers were weighed daily at 15:00 hours.

All experimental procedures were approved by Miyazaki Livestock Research Institute Animal Experimental Committee.

### 2.2 Feed intake, growth performance, blood metabolites, and ruminal profile

The feed intake was calculated by subtracting the amount of ort from the amount of feed provided daily. Every 2 weeks at 13:00 hours just after the morning feed (lasting 4 h), the body weight (BW) of the steers was measured.

After BW measurement, blood samples were collected in heparin‐containing sodium test tubes (Venoject II VP‐H100K; Terumo Co., Ltd., Tokyo, Japan) via a jugular vein puncture at −4, 0, 4, 8, and 12 weeks after the onset of the experiment. The tubes were immediately placed on ice in a light‐shielded box and were then centrifuged at 1,870 × *g* for 15 min at 4°C. Aliquots of plasma obtained from blood samples were transferred into micro tubes and were preserved it at −30°C until further analysis.

The ruminal fluid samples were aspirated after collecting blood samples using an oral tube (NFM90; Fujihira industry Co., Ltd., Tokyo, Japan) and were then filtered with four layers of sterile gauze. A portion of the filtered sample was processed as previously reported (Hirabayashi et al., 2017) to determine the activity of LPS. Another portion of the filtered sample was transferred to a vial and centrifuged at 1,840 × *g* for 10 min at 4°C. After centrifugation, the supernatant was collected in a screwcap vial and stored it at −30°C until analyzing it for VFAs and ammonia nitrogen (NH_3_‐N).

### 2.3 Feed digestibility, nitrogen balance, and daily changes in the pH and temperature of ruminal fluid

From weeks 4 to 8, after the onset of the experiment, the feed digestibility, nitrogen balance, and ruminal fluid pH and temperature of the steers were measured.

Feed digestibility and nitrogen balance were measured by collecting total feces and urine produced. The experimental period comprised a 7‐day adaptation period and 3‐day collection period. The urine sample was preserved by acidification, and the samples collected during the collection period were pooled and stored at 4°C. The samples were subsequently blended and analyzed.

The pH and temperature of the ruminal fluid were measured using a wireless radio transmission pH measurement system. The pH sensor had previously been developed principally for the academic research on SARA in dairy cattle (Kimura et al., 2012; Sato et al., 2012). The pH and temperature of the ruminal fluid were continuously measured every 10 min for 10 days between 6 and 8 weeks, after the onset of the experiment. The ruminal fluid pH and temperature data were summarized every 60 min.

### 2.4 Sample analyses

The experimental feed, ort, and fecal samples were dried for 1 day in a forced air oven at 60°C. The dried samples were ground through a 1‐mm screen using a Willy mill. The DM, crude protein (CP), ether extract (EE), crude ash, organic matter (OM), and nonfibrous carbohydrate (NFC) content were determined according to the conventional methods (Japan Grassland Agriculture and Forage Seed Association (GAFSA), 2009). The NDF content was measured using heat stable amylase and was expressed exclusive of residual ash (aNDFom; Van Soest, Robertson, & Lewis, 1991). The starch concentration was determined using the total starch assay kit (K‐TSTA‐100A; Megazyme, Wicklow, Ireland). The CP content in the fresh fecal and urine samples was also analyzed (Japan Grassland Agriculture and Forage Seed Association (GAFSA), 2009).

The concentration of plasma total protein (TP), albumin (Alb), total cholesterol (T‐Cho), triglycerides (TG), blood urea nitrogen, glucose (Glu), total ketone bodies (T‐KB), inorganic phosphorus (IP), and calcium (Ca), and the activity of aspartate transaminase (AST) were determined using HITACHI 7070 (Hitachi, Ltd., Tokyo, Japan). The plasma VA concentration was analyzed using HPLC L‐2130 (Hitachi, Ltd., Tokyo, Japan). The plasma growth hormone concentration was measured using radioimmunoassay (Hirabayashi et al., 2017). The plasma concentration of insulin (Ins) (AKRIN‐010T; FUJIFILM Wako pure chemical Corporation, Tokyo, Japan), LPS binding protein (LBP) (HK503 kit; Hycult Biotech, Uden, Netherlands), serum amyloid A (SAA) (TP‐802; Tri‐Delta Diagnostics Inc., Cedar Knolls, NJ, USA), and haptoglobin (Hp) (HAPT‐11; Life Diagnostics, Inc., PA , USA) was determined using commercially available enzyme‐linked immunosorbent assay kits.

The concentration of VFAs in the rumen fluid was analyzed using high‐performance liquid chromatography (CTO‐10AV; Shimadzu Corporation, Kyoto, Japan) via the bromothymol blue postlabel method. The concentration of NH_3_‐N in the rumen fluid was quantified using an ammonia kit (Ammonia‐Test; Wako Pure Chemical Industries, Ltd, Osaka, Japan). The activity of LPS in the rumen fluid was determined using the method of Hirabayashi et al. (2017).

### 2.5 Statistical analysis

The data were analyzed using the FIT model procedure of JMP^®^ (13.2.1; SAS Institute Inc., Cary, NC, USA). As one of the steers in the KP group presented decreased feed intake and DG due to illness, the data associated with it were excluded from statistical analyses.

The growth performance, feed digestibility, and nitrogen balance of the steers were analyzed using one‐way analysis of variance. The changes in blood and ruminal profiles or the pH and temperature of the ruminal fluid were analyzed using mixed model analysis. The following model was used:

$$Y_{\mathrm{ijk}}=\mu +\alpha_{i}+\beta_{\mathrm{ij}}+\gamma_{k}+\alpha \gamma_{\mathrm{jk}}+e_{\mathrm{ijk}}$$

where *Y*_ijk _= observation of dependent variables; μ = overall mean; α_i_ = effect of the dietary treatment *i*; β_ij_ = effect of the animal *j* with dietary treatment *i*; γ_k_ = effect of the period or time *k*; αγ_jk_ = interaction between the dietary treatment *i* and period or time *k*; and *e*_ijk_ = random error. The orthogonal contrasts were used to detect differences among specific treatments and periods. The contrasts included the control group versus the KP group; week −4 versus week 0, 4, 8, or 12 after of the onset of the experiment in the control group; and week −4 versus week 0, 4, 8, or 12 after the onset of the experiment in the KP group.

The results were considered significant if the *p* value was <0.05 in the F‐test.

## 3 RESULTS

The BW, DG, DM intake, and feed efficiency during the experimental period are presented in Table 2. There was no significant difference in the DG during the experimental period and the BW by the end of experiment in both the groups. There was no significant difference in the DM intake per metabolic body size (BW^0.75^) between the groups. The TDN intake per BW^0.75^ in the KP group tended to be lower (*p *<* *0.10) than that in the control group. The feed efficiency was not different between the groups.

**Table 2:** Effect of feeding kraft pulp (KP) on the growth performance of the Japanese Black fattening steers

Parameter	Control group	KP group	*SEM* a	*p*‐value
Number of steers	5	5	—	—
Growth performance				
Initial weight, kg	713.2	746.0	10.1	0.105
Final weight, kg	788.4	806.4	17.1	0.479
DG, kg/day	0.91	0.79	0.12	0.495
Feed intake^b^				
DM, g/BW^0.75^/day	59.1	55.0	2.0	0.180
TDN, g/BW^0.75^/day	49.9	45.2	1.3	0.063
Feed efficiency^b^				
DG/DM intake, g/kg	104.5	96.8	7.6	0.642
DG/TDN intake, g/kg	125.4	123.1	17.5	0.928

*Notes*. DG, daily gain; DM, dry matter; TDN, total disesrible nutrients; BW, body weight.

a Standard error of the mean.

b The data pertaining to one of steers in the KP group was excluded due to illness.

The nutrient digestibility of the Japanese Black fattening steers is shown in Table 3. There was no significant difference in the DM intake between the control and the KP groups. The DM, TDN, and CP intake per BW^0.75^ and concentrate feed ratio did not differ between the groups. The same applied to the digestibility of the DM, OM, CP, EE, aNDFom, NFC, and starch between the groups. Data pertaining to nitrogen balance are presented in Table 4. There was no significant difference between the control and KP groups in the ratio of feces N, urine N, and N retention.

**Table 3:** Effect of feeding kraft pulp (KP) on nutrient digestibility in the Japanese Black fattening steers

Parameter	Control group	KP group	*SEM* a	*p*‐value
Feed intake, kg/day				
DM	8.9	7.7	0.6	0.220
Feed intake, g/BW^0.75^/day				
DM	62.3	53.1	4.0	0.143
CP	8.2	6.8	0.6	0.143
TDN	51.3	44.6	3.1	0.167
Concentrate feed ratio^b^, %	92.3	90.0	1.5	0.300
Digestibility, %				
DM	81.2	83.5	1.4	0.270
OM	83.0	85.4	1.3	0.238
CP	73.9	76.6	1.8	0.310
EE	77.6	80.5	1.9	0.316
aNDFom	66.6	71.3	3.9	0.410
Non‐fibrous carbohydrate	92.0	94.9	1.3	0.158
Starch	97.5	98.9	0.7	0.191

*Notes*. DM, dry matter; BW, body weight; CP, crude protein; TDN, total digestible nutrients; OM, organic matter; EE, ether extract; aNDFom, α‐Amilas‐ treated ash‐free neutral detergent ifber.

a Standard error of the mean.

b The ratio of concentrate feed to dry matter intake.

**Table 4:** Effect of feeding kraft pulp (KP) on nitrogen balance in the Japanese Black fattening steers

Parameter	Control group	KP group	*SEM* a	*p*‐value
Ratio of nitrogen intake, %				
Feces N excretion	26.1	23.4	1.8	0.310
Urine N excretion	31.3	34.1	5.1	0.711
N retention	42.6	42.5	5.1	0.994

a Standard error of the mean.

**Figure 1:**
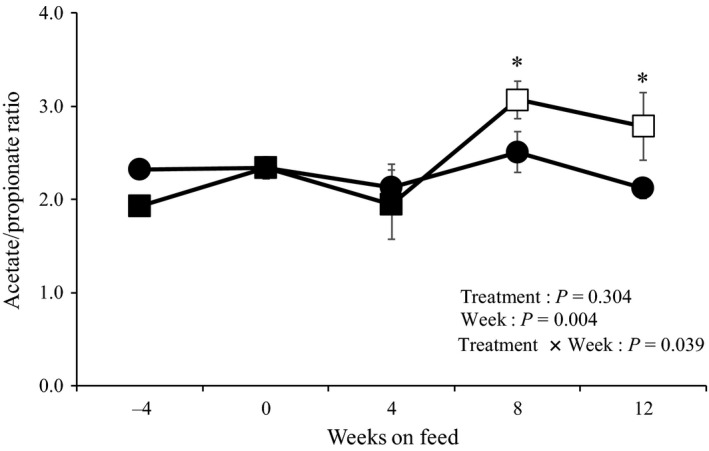
Changes in the acetate‐to‐propionate ratio in the rumen fluid in the control group (circles) and the KP group (squares) during the experimental period. The values are presented as mean ± *SEM*. White symbols indicate that the value is significant compared with that at week −4 (*p *<* *0.05). Data pertaining to one of the steers in the KP group were excluded due to illness in weeks 4 and 12. *The values are different from that of the control group steers at *p *<* *0.05

The ruminal profiles of the steers during the experimental period are presented in Table 5. The acetate‐to‐propionate (A/P) ratio in the rumen fluid is illustrated in Figure 1. There was no significant difference in the total VFA concentration in the ruminal fluid between the two groups. The ratio of acetic acid, propionic acid, and butyric acid in both the groups was not influenced by the diet. However, at weeks 8 and 12 after the onset of the experiment, the A/P ratio in the KP group was significantly higher than that in the control group (Figure 1). There was no significant difference in the concentration of ruminal fluid NH_3_‐N and activity of ruminal fluid LPS between the groups.

**Table 5:** Changes in the ruminal profile of the Japanese Black fattening steers in the control and KP group during the experimental period

Parameter	Group		*SEM* a	Weeks on feed					*SEM* a	*p*‐value		
	Control	KP		−4	0	4^b^	8	12^b^		Treatment	Week	Treatment × week
Total VFA^c^, mmol/L	88.9	91.1	2.8	94.3	81.5	99.6	94.9	79.7	3.8	0.603	0.001	0.997
Composition												
Acetic acid, %	54.8	55.4	0.7	54.6	55.1	53.3	58.7	53.7	1.1	0.545	0.009	0.258
Propionic acid, %	24.5	24.4	0.8	26.1	23.7	28.0	21.6	22.8	1.3	0.969	0.009	0.119
Butyric acid, %	16.2	15.9	0.7	15.9	16.8	13.8	15.1	18.6	1.0	0.790	0.031	0.460
Acetic:Propionic	2.3	2.4	0.1	2.1	2.3	2.0	2.8	2.5	0.1	0.304	0.004	0.039
Ammonia‐nitrogen, mg/dl	5.6	5.8	1.1	4.2	4.2	6.2	6.4	7.4	1.1	0.851	0.108	0.964
LPS^d^												
log_10_ EU/ml	4.89	4.71	0.08	4.87	4.58	5.16	4.77	4.61	0.10	0.155	0.001	0.163
EU/ml	77,814.5	50,786.4		74,418.1	38,051.6	146,919.6	58,524.7	40,322.6				

a Standard error of the mean.

b The data pertaining to one of steers in the KP group was excluded due to illness.

c Volatile fatty acid.

d Lipopolysacharide.

**Figure 2:**
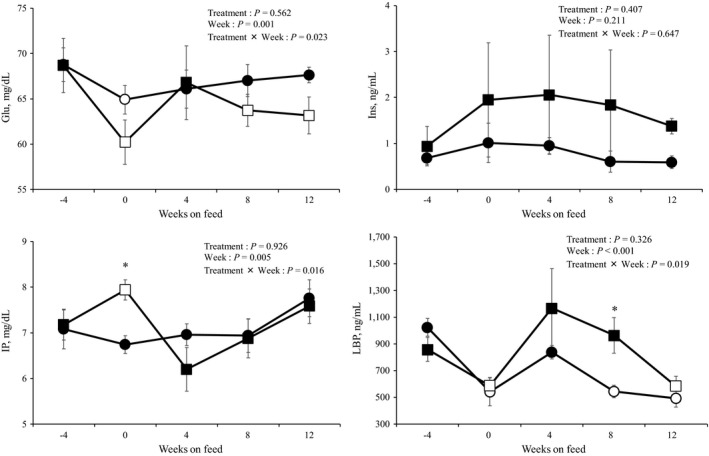
Changes in the plasma concentration of glucose (Glu), insulin (Ins), inorganic phosphorus (IP), and lipopolysaccharide binding protein (LBP) in the control group (circles) and the KP group (squares) during the experimental period. The values are presented as mean ± *SEM*. White symbols indicate that the value is significant compared with that at week −4 (*p *<* *0.05). The data pertaining to one of the steers in the KP group were excluded due to illness at weeks 4 and 12. *The values are different from that of the control group steers at *p *<* *0.05

The average daily ruminal fluid pH (control group vs. KP group = 6.36 vs. 6.08; *p *=* *0.172; data not shown) and temperature (control group vs. KP group = 38.7°C vs. 38.3°C; *p *=* *0.150; data not shown) were not affected by the KP diet. Diurnal changes in the ruminal fluid pH and temperature are illustrated in Figure 2. The lower ruminal fluid pH and temperature in the KP group than those in the control group throughout the experimental period was not statistically significant.

**Figure 3:**
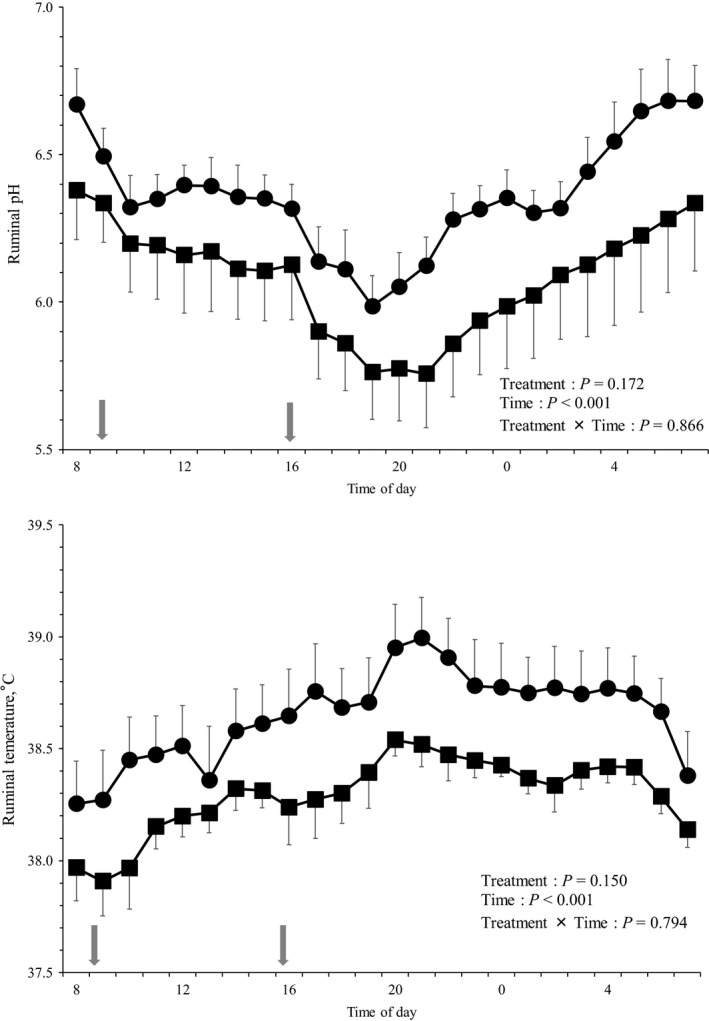
Diurnal changes in the ruminal pH and temperature of the Japanese Black fattening steers in the control group (circles) and the KP group (squares). The values are presented as mean ± *SEM*. Arrow: feed time. The data pertaining to one of the steers in the KP group were excluded due to illness

The blood components of the steers during the experimental period are presented in Table 6. There were no differences in the blood concentrations of plasma TP, Alb, albumin‐to‐globulin ratio, T‐Cho, TG, AST, blood urea nitrogen, Ins, growth hormone, T‐KB, Ca, VA, SAA, and Hp between the control and KP groups. The changes in the plasma concentrations of Glu, Ins, IP, and LBP during the experimental period are illustrated in Figure 3. The plasma concentration of Glu in both the groups significantly decreased at week 0 compared with that at week −4. There was no significant difference in the plasma concentration of Glu in the control group between week −4 and weeks 4, 8, and 12 after the onset of the experiment, respectively. In contrast, the plasma concentrations of Glu in the KP group at weeks 8 and 12 after the onset of the experiment, respectively, were significantly lower than that at week −4. However, there was no significant difference in the changes in the plasma Ins concentration between the control and KP groups. Furthermore, there was no significant difference in the plasma IP concentration in the control group between week −4 and weeks 0, 4, 8, and 12 after the onset of the experiment. The plasma IP concentration in the KP group at week 0 was significantly lower than that at week −4. At week 0, the plasma IP concentration in the KP group was significantly higher than that in the control group. The plasma LBP concentration in the control group significantly decreased at weeks 0, 8, and 12 after the onset of the experiment compared with that at week −4. The plasma LBP concentration in the KP group at weeks 0 and 12 after the onset of the experiment was significantly lower than that at week −4. At week 8 after the onset of the experiment, the plasma concentration of LBP in the KP group was higher (*p *< 0.05) than that in the control group.

**Table 6:** Changes in the blood profile of the Japanese Black fattening steers in the control and KP group during the experimental period

Parameter	Group		*SEM* a	Weeks on feed					*SEM* a	*p‐*‐value		
	Control	KP		−4	0	4^b^	8	12^b^		Treatment	Week	Treatment × week
TP, g/dl	7.6	7.9	0.2	7.8	7.9	6.9	7.4	8.7	0.3	0.304	0.002	0.492
Alb, g/dl	3.6	3.9	0.1	3.7	3.9	3.2	3.5	4.3	0.1	0.065	<0.001	0.982
A/G ratio	0.95	0.99	0.05	0.94	1.01	0.98	0.92	0.99	0.05	0.548	0.177	0.466
T‐Cho, mg/dl	164	154	10	164	184	130	141	179	10	0.491	<0.001	0.635
TG, mg/dl	12	11	1	12	11	11	10	13	1	0.462	0.179	0.114
AST, U/L	69	72	5	70	71	61	67	81	5	0.673	0.096	0.552
BUN, mg/dl	12.0	13.6	0.7	11.6	13.3	11.7	11.8	15.6	0.8	0.151	0.002	0.658
Glu, mg/dl	66.9	64.5	2.3	68.7	62.6	66.4	65.4	65.4	1.6	0.434	<0.001	0.044
Ins, ng/ml	0.77	1.63	0.70	0.81	1.48	1.51	1.22	0.98	0.54	0.407	0.211	0.647
GH, ng/ml	2.46	2.69	0.22	3.04	2.44	2.55	2.42	2.43	0.30	0.490	0.487	0.221
T‐KB, mg/dl	397.3	372.5	25.0	339.1	381.3	376.7	431.2	396.2	26.8	0.504	0.079	0.898
IP, mg/dl	7.1	7.2	0.7	7.1	7.3	6.6	6.9	7.7	0.3	0.867	0.022	0.045
Ca, mg/dl	10.5	11.1	0.2	10.7	11.0	9.6	10.5	12.3	0.3	0.142	<0.001	0.337
VA, IU/dl	55	51	3	44	48	59	57	57	5	0.295	0.146	0.491
LBP, ng/ml	687.1	789.1	177.6	940.6	562.7	894.5	753.7	539.0	71.1	0.326	<0.001	0.019
SAA, μg/ml	19.83	18.73	4.14	33.52	13.54	30.37	9.67	9.32	4.43	0.856	<0.001	0.649
Hp, μg/ml	1.97	7.07	3.17	7.50	0.96	9.06	0.94	4.14	3.16	0.288	0.087	0.220

TP, total protein; Alb, albumin; A/G, albumin/globulin; T‐Cho, total cholesterol; TG, triglycerides; AST, aspartate transaminase; BUN, blood urea nitrogen; Glu, glucose; Ins, insulin; GH, growth hormone; TKB, total ketone bodies; IP, inorganic phosphorus; Ca, calcium; VA, vitamin A; LBP, lipopolysaccharide binding protein; SAA, serum amyloid A; Hp, haptoglobin.

a Standard error of the mean.

b The data pertaining to one of steers in the KP group was excluded due to illness.

## 4 DISCUSSION

In this study, the DM intake was unaffected by the addition of KP, and the growth performance and feed efficiency were comparable between the groups. Several diverse reports on the effects of additive wood‐based feed on feed consumption and growth arrive at inconsistent conclusions regarding the effect on growth, which appears to depend on whether the wood‐based feed replaces the concentrate feed or the roughage. No effect on the DM intake or DG was observed when the steam‐treated wood‐based feed was supplied to Holstein steer as a substitute for alfalfa hay cubes (Kajikawa et al.,^1987^). In contrast, when the sulfite‐treated wood‐based feed was supplied to beef cows instead of barley, no effect was observed on the DM intake but the DG decreased (Clarke & Dyer, 1973). In this study, the addition of KP was not found to affect the DM intake; which is in agreement with the findings of the previous report. However, unlike in the previous report, replacing concentrate feed with KP was not found to affect the DG in this study. It is considered that the digestibility of wood‐based feed influenced the variance between the results.

The digestibility of wood‐based feed changes depending on the processing method of wood (Baker, 1973; Millett, Baker, Satter, McGovern, & Dinius, 1973). Baker (1973) indicated that an increase in the lignin removal rate from 0% to 96.5% increased the in vitro DM digestion rate from 12% to 90%. The lignin content and the DM digestion rate of steam‐treated wood‐based feed are 13.0% and 60.6%, respectively (Takigawa, 1987). The lignin content of alkali‐treated KP was reduced to no more than 5%, whereas the in situ DM digestion rate was at least 90% (Hada et al., 2016). In this study, the addition of KP was not found to affect the digestion rate of each feed component or nitrogen utilization. Kraft pulp is more digestible than conventional wood‐based feeds, and its digestion rate is comparable with that of concentrate feed. In consequence, feeding KP instead of concentrate feed to the Japanese Black fattening steer is not considered to affect their growth or feed efficiency. Furthermore, no effect on feed digestibility was observed in lactating cows even when 12% of KP was admixed into the feed instead of rolled corn (Nishimura et al., in press).

The levels of blood biochemical components observed in this study were within the clinically normal ranges for the Japanese Black fattening steer (Adachi et al., 1999; Kaneko, 1983; Nakamura et al., 2008). The addition of KP was found to affect the plasma concentrations of Glu and IP. Ruminants depend on gluconeogenesis in the liver for endogenous Glu, and one of the substrates of gluconeogenesis is propionic acid (Young, 1977). In this study, the A/P ratio in the rumen fluid of steers in the KP group was significantly higher than that in the control group at weeks 8 and 12, which was considered to be associated with the simultaneous decrease in the plasma Glu concentration in the KP group. The plasma IP concentration is related to carbohydrate metabolism (Kaneko, 1983). The plasma IP concentration may rise on the fasting (Kaneko, 1983). However, in this study, there was no significant difference in the DM intake and concentrate feed ratio between the groups. Therefore, there was no clear relationship between plasma IP concentration and carbohydrate metabolism in the KP group.

The A/P ratio in the rumen fluid increased with the addition of KP. The same increase in the A/P ratio was obtained when the wood‐based feed was provided as a substitute for concentrate feed (Clarke & Dyer, 1973; Nishimura et al., in press). The substitution with the wood‐based feed decreases the proportion of starch and increases the proportion of NDF consumed, thereby increasing the proportion of acetic acid in VFAs. In previous study, replacing 50% of rolled corn DM with KP increased the proportion of NDF (control group vs. KP group = 39.6% vs. 47.0%), decreased the proportion of starch (control group vs. KP group = 28.7% vs. 19.7%), and increased acetic acid ratio and decreased the propionic acid ratio in VFAs (Nishimura et al., in press). In our study, no significant difference was observed between the control and KP groups in the proportion of aNDFom consumed (24.5% vs. 27.0%; *p* = 0.467; data not shown). However, the proportion of starch consumed decreased significantly in the KP group compared with that in the control group (45.9% vs. 48.8%; *p* = 0.035; data not shown). This might have affected the A/P ratio in the steers. There was no dietary treatment effect or interaction between the dietary treatment and period in acetic acid ratio in our study. However, the acetic acid ratio in the control and KP groups was 57.7% versus 59.6% at 8 weeks after the onset of the experiment and 51.8% versus 55.7% at 12 weeks after the onset of the experiment (data not shown). The propionic acid ratio in the control and KP groups was 23.6% versus 19.7% at 8 weeks after the onset of the experiment and 24.6% versus 21.0% at 12 weeks after the onset of the experiment (data not shown). Therefore, it is also considered that the change in the A/P ratio in the KP group in this study was influenced by two factors: an increase in the acetic acid ratio and decrease in the propionic acid ratio.

An increase in the feed starch content in feed decreases the pH and increases the LPS activity in the ruminal fluid (Plaizier, Khafipour, Li, Gozho, & Krause, 2012). In this study, the concentrate feed comprised at least 90% of the consumed feed in both the groups and aNDFom comprised no more than 30%; no significant difference was found between the groups. Zebeli, Metzler‐Zebil, and Ametaj (2012) reported that if the proportion of concentrate feed consumed by dairy cattle is at least 35% or if the proportion of NDF in the feed is 44.7% or less, the LPS activity in the rumen fluid will increase. As no findings pertaining to SARA in the Japanese Black fattening steer have been reported, the breakpoint of the factors affecting the ruminal fluid pH and LPS activity remain unclear. Furthermore, during the fattening of Japanese Black steer, the proportion of the supplied concentrate feed ranges from 80% to over 85% (National Agriculture and Food Research Organization (NARO), 2009), and therefore, it is difficult to compare the findings with those of the dairy cattle (Zebeli et al., 2012). In this study, the substitution of 10% of the concentrate feed with KP had an effect on the amount of starch and aNDFom consumed. However, steers still intake large quantities of starch and small quantities of NDF. This was considered to be the reason for no effect on the ruminal fluid pH or LPS activity. In future, the effect of KP addition during the early period (when the proportion of concentrate feed supplied is low) and middle period of fattening (when the roughage‐to‐concentrate ratio in the feed supplied fluctuates) both need to be further explored.

The death of microorganisms and production of LPS, which is a component of bacteria, are promoted by a decrease in the pH of ruminal fluid due to the oversupply of concentrate feed (Plaizier et al., 2012). The LPS released into the gastrointestinal tract is transferred to the blood vessels, where it might cause a systemic inflammatory reaction (Plaizier et al., 2008, 2012). In several studies, the LPS activity in the ruminal fluid and the plasma concentration of acute phase proteins (SAA, Hp, and LBP) are mutually related and are considered as inflammatory markers. For instance, a decrease in the ruminal fluid pH increases the activity of LPS in the ruminal fluid, and the concentration of SAA (Gozho, Krause, & Plaizier, 2006; Gozho et al., 2005; Khafipour, Krause, & Plaizier, 2009a), Hp (Gozho et al., 2005, 2006; Khafipour et al., 2009a), and LBP (Khafipour et al., 2009a) in the blood. However, it has also been reported that an increase in the activity of LPS in the ruminal fluid does not affect the blood concentration of the SAA, Hp, and LBP (Khafipour, Krause, & Plaizier, 2009b; Plaizier et al., 2014). These inconsistent findings regarding the relationship between LPS activity in the ruminal fluid and acute‐phase protein concentrations in the plasma may be due to the differing individual responses of rumen fluid LPS activity to the same feed (Hirabayashi et al., 2017). Furthermore, the reason for the transfer of ruminal fluid LPS to the bloodstream remains unclear. In this study, the addition of KP was not found to affect the activity of LPS in the ruminal fluid or the concentration of SAA and Hp in the plasma. At week 8, the plasma LBP concentration in the KP group increased significantly compared with that of the control group. It was conjectured that this was influenced by feeding KP as well as due to the differing individual responses.

The administration of KP as a substitute for a portion of concentrate feed exhibited no effect on the growth or feed efficiency of the Japanese Black fattening steer. Additionally, the addition of KP increased the A/P ratio in the rumen fluid, without affecting the ruminal fluid pH or LPS activity. The effect of increasing the proportion of KP addition during the early and middle phases of fattening needs to be further investigated.
